# Artificial Intelligence Across the Radiology Workflow: A Nine-Stage Narrative Review

**DOI:** 10.3390/diagnostics16101485

**Published:** 2026-05-13

**Authors:** Marwa Chendeb El Rai, Aicha Beya Far, Muna Darweesh, Salam Dhou, Nour Aburaed, Salah El Rai, Mohammed ElKhazendar, Samer Ellahham

**Affiliations:** 1 Mathematics Division, American University in Dubai, Dubai 28282, United Arab Emirates; 2 Department of Electrical and Computer Engineering, American University in Dubai, Dubai 28282, United Arab Emirates; afar@aud.edu; 3 College of Engineering and Information Technology, University of Dubai, Dubai 14143, United Arab Emirates; midarweesh@ud.ac.ae (M.D.); nour.aburaed@ieee.org (N.A.); 4 Department of Computer Science and Engineering, American University in Sharjah, Sharjah 26666, United Arab Emirates; 5 Department of Radiology, Dr. Sulaiman Al Habib Hospital, Dubai 505005, United Arab Emirates; salah_raii@yahoo.fr; 6 Department of Internal Medicine, Sheikh Khalifa Medical City, Abu Dhabi 289143, United Arab Emirates; 7 Heart, Vascular & Thoracic Institute, Cleveland Clinic, Abu Dhabi 112412, United Arab Emirates

**Keywords:** artificial intelligence, radiology workflow, medical imaging, large language models, clinical deployment, workflow optimization, narrative review

## Abstract

Radiology services are experiencing increasing operational complexity due to rising imaging volumes and expanding coordination demands across interconnected clinical and administrative processes. This complexity is reflected in variability across workflow stages, driven by fragmented information flows, heterogeneous system integration, and multi-source data dependencies. Artificial intelligence (AI) has therefore emerged as a potential tool to support automation, prioritization, and operational efficiency throughout the radiology pathway. This narrative review examines published applications of AI within a nine-stage representation of the radiology workflow. The review synthesizes how AI methods are being investigated to support both administrative coordination and diagnostic processes in radiology practice. AI approaches aim to reduce repetitive administrative tasks, improve resource utilization, and assist radiologists in managing increasing imaging workloads. However, research activity remains uneven, with a strong concentration on later-stage tasks such as image analysis and reporting, while earlier and administrative stages remain comparatively underexplored. By organizing existing research within a unified workflow-oriented framework, this review highlights areas of concentration and identifies gaps across less-studied stages. The findings suggest that while several AI applications are approaching early clinical deployment, broader workflow-level impact remains limited by challenges related to system integration, interoperability, governance, and real world implementation. Continued progress will depend on developing integrated and clinically validated solutions that extend beyond isolated tasks to support coordinated radiology workflow optimization.

## 1. Introduction

The demand for radiology services continues to grow, with examinations becoming more complex and expectations for diagnostic accuracy steadily increasing [1,2,3]. Radiology workflows are characterized by regular transitions between tasks, fragmented information flow, and repeated context transitions, which may affect efficiency and cognitive workload in high-throughput clinical settings. The radiology workflow functions as an interdependent value chain in which inefficiencies arising at early stages may propagate downstream, amplifying operational friction across interconnected processes. Addressing workflow fragmentation has therefore prompted increasing interest in system-level approaches focused on process integration, task coordination, and information continuity [4,5]. Within this context, Artificial Intelligence (AI) has been investigated as a tool to support diagnostic, administrative, and coordination-related processes across the radiology pathway [6,7,8]. Workforce capacity and imaging demand have been described as misaligned in several regions, particularly following pandemic disruptions [9], although evidence suggests that frequent AI use may paradoxically increase radiologist burnout risk, particularly under high workload conditions [10,11]. Although AI is frequently associated with image anomaly detection, published applications extend beyond diagnostic interpretation to include operational coordination and workflow optimization across multiple stages of the radiology pathway [7,8]. However, the distribution of published studies across workflow stages is uneven. A substantial proportion of the literature concentrates on downstream diagnostic activities, particularly image analysis, interpretation, and report generation. In contrast, several operational stages remain comparatively less represented in published research, including pre-approval and billing processes, scheduling and patient flow management, and image acquisition or post-processing. Upstream clinical decision-making processes such as exam ordering, as well as downstream activities related to patient engagement and follow-up, are also addressed in a smaller number of studies. This distribution reflects the historical emphasis of AI research on image interpretation tasks, while administrative coordination and workflow management processes have received comparatively less attention in the radiology literature. Recent AI applications rely primarily on Machine Learning (ML) and Deep Learning (DL), employing computer vision (CV) methods [12,13] such as Convolutional Neural Networks (CNNs), Generative Adversarial Networks (GANs), and graph-based approaches for classification, detection, and segmentation tasks [14,15]. Natural Language Processing (NLP), including transformer-based models such as Bidirectional Encoder Representations from Transformers (BERT), Generative Pre-trained Transformer (GPT), and large language models (LLMs), has expanded automated processing of unstructured clinical text and administrative data [16,17]. Performance reporting commonly includes metrics such as area under the curve (AUC), accuracy, F1 score, and turnaround time (TAT) [6,18,19]. Despite these advances, existing reviews often focus on specific algorithmic approaches, imaging modalities, or selected segments of the radiology workflow rather than examining the entire administrative and clinical pathway. The nine-stage workflow model used in this review was derived through comparative synthesis of previously published radiology workflow taxonomies. Table 1 illustrates how stage boundaries described across representative frameworks correspond to the stages adopted in this study. Where prior frameworks grouped multiple operational processes within a single stage, the present model separates them when the literature demonstrates distinct categories of AI applications or operational decision points. Existing taxonomies typically include ordering, protocoling, acquisition, interpretation, and reporting; however, billing, pre-approval, and patient follow-up are inconsistently represented across prior decompositions [20,21,22,23,24]. This narrative review synthesises published applications of AI across a nine-stage representation of the radiology workflow derived from a convergent synthesis of previously published workflow taxonomies [20,21,22,23,24,25,26]. The framework is used as an organisational structure for reviewing the literature rather than as a proposed theoretical model of radiology workflow. Although prior workflow descriptions focused on specific algorithmic domains, imaging modalities, or selected segments of the radiology workflow [20,23,27,28,29], the present review includes stages such as pre-approval and billing (Stage 2) and patient engagement (Stage 9) to capture the full administrative and clinical pathway represented in the literature. The nine stages considered in this review are: (1) Clinical Test Ordering and Exam Selection; (2) Pre-approval Process and Billing; (3) Scheduling and Patient Flow Management; (4) Exam Protocoling, Patient Positioning, and Dose Optimization; (5) Image Acquisition and Post-processing; (6) Radiologist Assignment and Worklist prioritization; (7) Image Analysis and Interpretation; (8) Report Generation and Communication; and (9) Follow-up and Patient Engagement.

The nine-stage representation therefore provides a structured lens through which reported AI applications can be synthesised across radiology practice. By situating these applications within an interconnected operational framework, the review examines how published studies address different components of radiology practice, including decision support at ordering, acquisition optimization, interpretation assistance, structured reporting, communication, and follow-up coordination. This perspective enables comparison of how AI applications are distributed across workflow stages and highlights areas where research activity remains limited. Beyond descriptive synthesis, this review highlights structural imbalance in evidence distribution and cross-stage dependencies that constrain real world AI deployment. The remainder of the paper is organised as follows. Section 2 presents the narrative review methodology used to identify and synthesise relevant studies. Section 3 introduces the nine-stage workflow framework and outlines the operational context in which AI systems are deployed. Subsequent sections examine reported AI applications across the workflow stages, followed by a discussion of cross-stage integration challenges, implementation constraints, and remaining gaps in the literature. The concluding section summarises the principal findings and outlines directions for future research on AI-supported radiology workflow optimization.

## 2. Narrative Review Methodology

**Figure 1:**
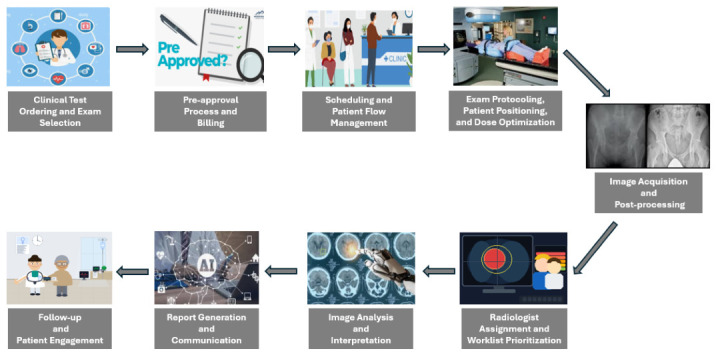
Stage-based overview of the radiology workflow, illustrating the sequential clinical and operational steps used as a reference framework for AI applications discussed in this review.

This review adopts a narrative rather than systematic approach. The search focused on identifying representative studies with evidence across each workflow stage. This work examines published research on the integration of AI across the radiology workflow, with the aim of providing a conceptual and operational overview of how AI applications interact with routine radiology processes. The review therefore characterizes how reported AI systems contribute to workflow optimization across nine interconnected operational stages defined in the Introduction and summarised in Table 1 and Figure 1. Within this framework, the review examines how AI applications influence diagnostic performance, image quality, reporting efficiency, operational coordination, and patient engagement. Search strategies combined AI-related descriptors such as artificial intelligence, machine learning, deep learning, natural language processing, and optimization with radiology workflow terminology and imaging-related terms, including CT, MRI, X-ray, ultrasound, and PET. Additional task-oriented keywords were used to capture workflow stages, including clinical ordering, scheduling, protocoling, image acquisition, worklist prioritization, interpretation, reporting, and patient follow-up. Search terms were not restricted to specific algorithmic architectures, enabling retrieval of studies spanning successive generations of AI development, from traditional machine learning and convolutional neural network approaches to transformer-based models, large language models, and emerging agentic systems. The distribution of architectural approaches within each workflow stage therefore reflects the composition of the published literature rather than a deliberate generational selection criterion. The reviewed studies were not systematically assessed for data partitioning practices. Datasets containing multiple consecutive cross-sections from the same patient require strict patient-level separation of training, validation, and test sets to prevent data leakage, which can artificially inflate reported performance metrics [30]. This consideration is particularly relevant to the high accuracy and AUC values cited across image analysis and interpretation tasks. Eligibility criteria prioritised studies reporting empirical evaluation of AI systems within clinical or operational radiology contexts. Literature searches were conducted across PubMed, Scopus, and Web of Science. Included studies were peer-reviewed full-text articles published in English between January 2015 and March 2026. Eligible studies reported operational or clinical performance endpoints such as prioritization accuracy, reporting efficiency, radiation dose reduction, image quality improvement, time-based workflow metrics, or patient communication outcomes. Studies conducted within hospital administrative systems were included when their outputs directly influenced radiology workflow processes.

Editorials, opinion articles, commentaries, letters, and conference abstracts without accompanying peer-reviewed full-text publications were excluded. Animal research, phantom-only evaluations, and purely simulated experiments without human clinical data were not considered. AI applications developed exclusively outside radiology clinical environments were similarly excluded unless they addressed administrative processes directly supporting radiology services.

Each publication was categorized according to the primary stage of the radiology workflow at which the AI system interacts with routine clinical operations. When an investigation addressed multiple workflow stages, it was assigned to the stage representing the principal evaluative focus of the reported application. Publications within each stage were synthesised thematically to describe how AI applications influence operational performance across tasks such as protocol selection, image acquisition, diagnostic interpretation, worklist prioritization, report generation, and patient communication. Information regarding study context, validation setting, and implementation environment was documented to provide context for reported performance metrics. Across the reviewed literature, study designs vary substantially and include single-institution evaluations, feasibility studies, simulation-based assessments, and a smaller number of clinical deployment reports. These methodological differences are noted within stage-specific discussions to contextualise the reported results, which should therefore be interpreted primarily as indicators of task-level feasibility rather than definitive evidence of routine clinical deployment. To situate reported evidence within a reproducible developmental reference, this review adopts the Technology Readiness Level (TRL) scale as a supplementary instrument for characterising the maturity of AI applications across workflow stages. Originally developed by NASA and subsequently adapted for biomedical and health informatics contexts, the TRL scale provides a nine-level taxonomy progressing from basic principle observation and proof-of-concept validation (TRL 1–3, Research Phase), through validation in relevant clinical environments (TRL 4–5, Validation Phase), to prototype demonstration in operational radiology settings (TRL 6–7, Prototype Phase), and finally to full certification and routine clinical deployment (TRL 8–9, Deployment Phase). This framework is applied to contextualise the maturity of reported AI applications across the nine workflow stages [25,26,31].

## 3. Artificial Intelligence Integration in the Radiology Workflow

AI approaches have been developed to improve automation and operational efficiency across multiple stages of the radiology workflow. Rather than operating solely as diagnostic tools, these systems increasingly support workflow coordination and decision-making from initial clinical assessment through reporting and follow-up. For example, NLP applied to Electronic Health Records (EHRs) can assist in appropriate imaging selection and guideline adherence [32], while predictive analytics has been used to optimise scheduling, patient flow, modality utilization [33], acquisition optimization and real-time protocoling during image capture [34]. In downstream interpretive stages, DL algorithms have demonstrated strong performance in detection and diagnostic support for complex conditions such as pulmonary nodules, breast cancer, and intracranial haemorrhage [35,36,37]. AI can also contribute to operational management by enabling dynamic worklist prioritization, allowing urgent examinations to be escalated for timely radiologist review [38]. Following image interpretation, AI methods support post-analytical processes including automated report structuring, information extraction, and communication of findings to referring clinicians and patients [39]. The following subsections examine reported AI applications across each stage of the radiology workflow, highlighting current areas of clinical implementation and aspects requiring further validation.

### 3.1. Clinical Test Ordering and Exam Selection

**Figure 2:**
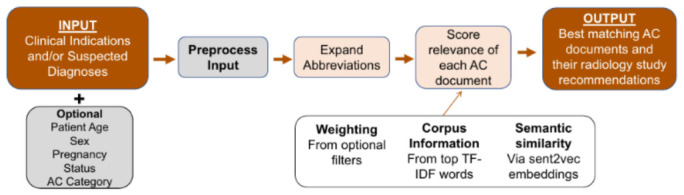
Overview of an NLP Algorithm for Matching Clinical Indications to Appropriateness Criteria Guidelines [32]. CC BY 4.0.

**Figure 3:**
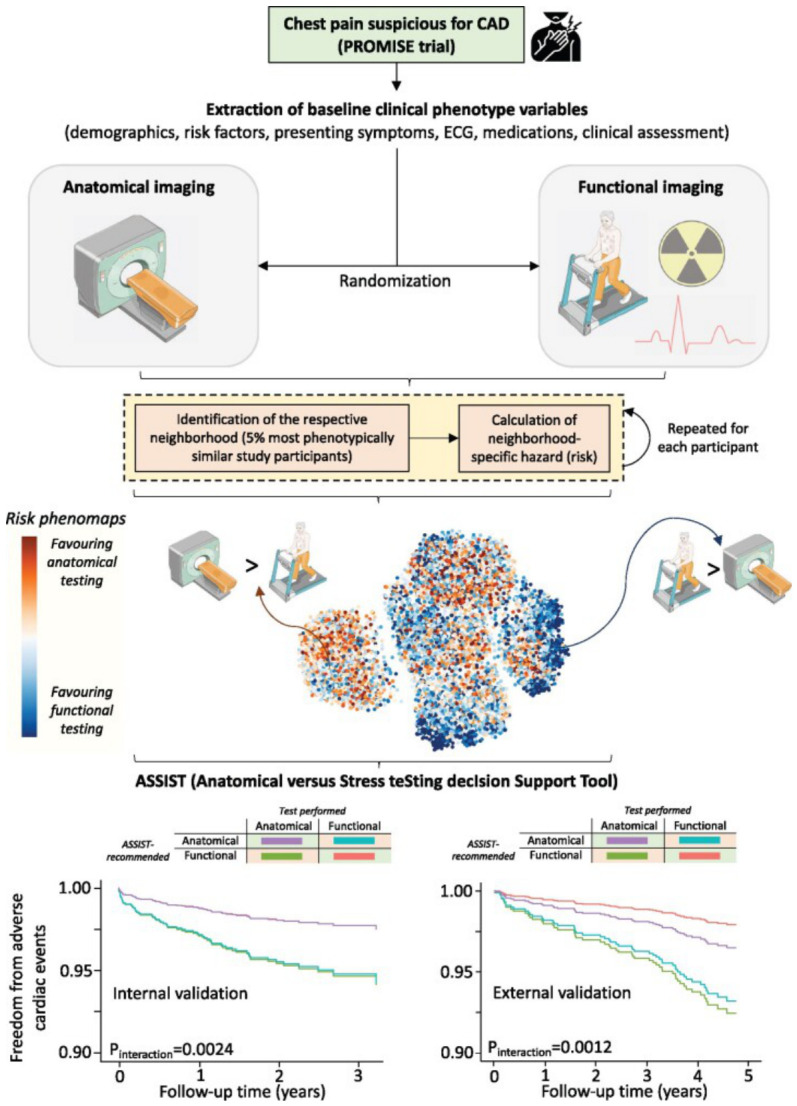
ASSIST personalizes imaging test selection for coronary artery disease patients [42]. Reproduced with permission from Oxford University Press.

Diagnostic imaging workflows begin with test ordering, where clinical indications, guideline adherence, and administrative requirements intersect. Incomplete referrals and fragmented documentation often require radiologists to reconstruct the clinical context from multiple unstructured notes, increasing decision complexity under high workload conditions [40]. These limitations have made the ordering stage an early focus for AI-enabled clinical decision support (CDS), particularly as appropriateness criteria and insurance authorization requirements continue to evolve. At this stage, AI systems function as CDS tools, using NLP and ML to extract indications from free text EHRs and align them with the modality appropriateness criteria [40]. Most studies report improvements in documentation structure and guideline concordance rather than fully automated decision-making. Large scale evaluations report measurable operational gains. A multicenter deployment analyzing 266,029 outpatient imaging orders increased structured order entry from 34.6% to 67.3% and appropriateness scoring from 30% to 52% following AI-assisted analysis, although nearly half of orders remained unscored [41]. Similarly, validation of the American College of Radiology NLP based CDS tool showed that the correct guideline appeared within the top three results for 98% of simple queries, 85% of complex queries, and 86% of real world indications [32], reflecting reliable retrieval of structured indications (Figure 2). Beyond guideline matching, predictive approaches can introduce greater personalization into exam selection. In [42], the ASSIST framework, derived from 9572 patients in the PROMISE trial, estimated individualized benefit from anatomical versus functional testing; alignment with model recommendations was associated with lower major adverse cardiovascular event rates (interaction *p*-values 0.0024 and 0.0321). This illustrates a shift from rule-based guideline matching toward risk-stratified imaging selection, as shown in Figure 3. Prospective adoption analyses further identify interoperability and governance barriers as continuing challenges for implementation [43]. Across the reviewed literature, study designs include retrospective operational analyses [41], NLP retrieval evaluations [32], externally validated predictive models [42], and prospective implementation studies [43]. However, prospective multi-site evaluation of fully integrated AI-assisted ordering systems within routine radiology workflows has not yet been reported. Key limitations include dependence on documentation quality [21], reduced performance for vague indications [32], incomplete scoring coverage [41], post hoc model development [42], and interoperability and organizational barriers affecting real world deployment [43]. Table 2 summarises representative contributions and limitations. Overall, the evidence indicates that AI deployment at this stage remains at an early clinical readiness level rather than full operational maturity, corresponding to TRL 4–5 [25,26,31].

### 3.2. Pre-Approval Process and Billing

Published research papers on AI methods used in pre-approval and billing in radiology remain limited despite comprehensive database searches. The scarcity of studies reflects structural characteristics of healthcare reimbursement systems, including cross national variation in billing frameworks, regulatory and liability constraints surrounding automated coding decisions. The pre-approval and billing stage introduces substantial administrative complexity in radiology workflows. Manual coding accuracy ranges from 50% to 98% depending on coder experience and case complexity [44], and inefficiencies at this stage are estimated to contribute to 15–25% of healthcare expenditure waste while propagating downstream delays in reporting and coordination [21]. AI applications at this stage mainly focus on automated coding and administrative data extraction. NLP and ML approaches demonstrate technical feasibility but remain constrained by document redundancy, evolving coding systems, and the difficulty of formalizing implicit billing logic [44]. The large language models have shown improved coding efficiency compared with the general models [45]. An NLP system applied to 200 craniospinal CT and MRI reports achieved sensitivity 0.88, specificity 0.80, and F2 0.83 for the top-5 ICD-10 (International Classification of Diseases, Tenth Revision) codes, with neuroradiologist inter-rater agreement ranging from Krippendorff α=0.39–0.63 [46]. Reimbursement systems for imaging services were not designed for AI-augmented workflows, creating uncertainty in coding and payment pathways [47,48,49,50]. At the system level, large-scale claims data indicate that radiologist billing for AI-supported services has increased substantially in recent years, although reimbursement pathways remain structurally misaligned with AI-augmented workflow models [51]. Operational evidence remains limited. An NLP pipeline tracking overdue follow-up recommendations demonstrated revenue recovery within a radiology department [52]. Policy analyses report high overturn rates for denied reimbursement decisions [53] and suggest that algorithmically driven denials may disproportionately affect vulnerable populations [54], highlighting governance and equity concerns. Across the reviewed corpus, evidence derives mainly from retrospective coding evaluations [46], single-institution deployments [46,52,55,56], and policy analyses [53,54]. Prospective multi-payer validation of integrated AI-assisted coding has not yet been reported. Limitations include performance decline beyond the most frequent codes [55], single institution feasibility without multi-payer validation [56], and governance concerns in algorithmically driven denial systems [53,54]. Table 3 summarises representative contributions and limitations. Most reported results derive from single-institution feasibility studies with limited coding scope, and cross-institutional or multi-payer validation remains lacking. Overall, AI applications at this stage remain at the feasibility level rather than approaching operational deployment, corresponding to TRL 2–3 [25,26,31].

### 3.3. Scheduling and Patient Flow Management

Radiology scheduling represents a key operational constraint in which resource allocation, modality availability, and unpredictable patient attendance influence throughput and downstream reporting timelines [20,57]. No-shows and late cancellations generate financial losses and propagate delays across the workflow chain [58]. These challenges are particularly important in high-throughput departments, where idle time accumulates across modalities and shifts, motivating interest in AI-based predictive tools to improve patient flow and resource utilization. ML no-show prediction is the most studied AI application at this stage. Electronic Medical Record (EMR) variables including scheduling to appointment interval, insurance type, and prior no-show history have demonstrated predictive value, with logistic regression achieving AUC 0.77 across 54,652 radiology appointments [59]. Gradient Boosted Trees trained on 4.5 million outpatient imaging visits achieved retrospective AUC 0.93 and prospective AUC 0.73 [58]. In [60], an AI predictive analytics system reduced outpatient appointment no-shows through targeted patient outreach for MRI scheduling. The evaluation was limited to a single institution using a retrospective pre-post design. Beyond attendance prediction, ML models applied to Radiology Information Systems (RIS) data have been used to forecast patient wait times and appointment delays across CT, MRI, ultrasound, and radiography. In these studies, modality, time of day, and delays from prior patients were identified as key predictive features [61]. Authors of [62] described a continuous learning AI framework embedded within radiology operations that adapts to evolving workflow patterns such as scanner utilization, patient throughput, and technologist variability. Operational deployment is constrained by proprietary data silos that limit interoperability across legacy RIS platforms [20] and by automation bias when predictions are applied without appropriate human oversight [57]. Models trained on historically stable scheduling patterns may underperform during periods of workflow disruption, highlighting the need for continuous retraining and mechanisms that allow human override. Across the literature, study designs include retrospective predictive modeling [59,61], limited prospective validation [58], single institution deployments [60], and conceptual operational frameworks [62]. No prospective multi-site evaluation of integrated AI-assisted scheduling systems has been reported. Key limitations include single institution scope [60], short validation periods [58], dependence on RIS data quality and platform constraints [20], and lack of prospective outcome studies linking prediction accuracy to measurable workflow improvements [62]. Table 4 summarises representative contributions and limitations. Overall, AI applications for radiology scheduling are approaching clinical readiness for prediction tasks such as no-show forecasting but remain insufficiently validated for fully integrated autonomous systems, corresponding to TRL 4–5 [31].

### 3.4. Exam Protocoling, Patient Positioning, and Dose Optimization

**Figure 4:**
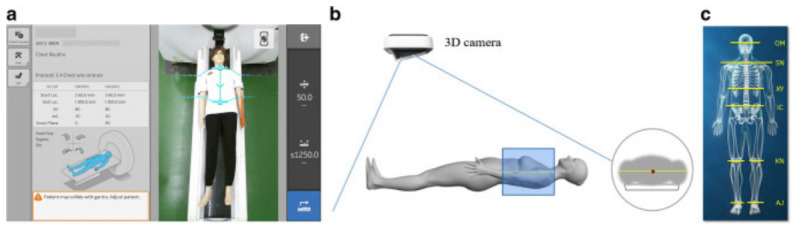
AI-based automatic CT patient positioning. (**a**) Auto-positioning scanner interface. (**b**) 3D camera-based depth detection for table height alignment. (**c**) Eight anatomical landmark references: OM, orbital meatal baseline; SN, sternoclavicular notch; XY, xyphoid; IC, iliac crest; KN, knee; AJ, ankle joint [69]. CC BY 4.0.

Exam protocoling, patient positioning, and scan triggering determine acquisition parameters, anatomical alignment, and timing, directly influencing image quality, radiation exposure, and repeat examination rates [63,64]. Errors at this stage propagate downstream, particularly in CT, where patient body habitus and scanner heterogeneity contribute to vertical centering errors and protocol deviations [64]. The COVID-19 pandemic further highlighted the importance of safety in minimizing the contact of technologist with the patient during positioning [65,66]. AI applications support protocol selection, positioning assistance, landmark detection, and dose optimization. In emergency brain MRI protocoling [67], a DL model achieved 84% protocol accuracy and 91% contrast agent accuracy, comparable to non subspecialty radiologists. Because positioning errors remain a major source of repeat imaging and dose inefficiency, AI for positioning has been proposed to improve centering consistency and reduce contact intensive adjustments [68,69] as illustrated in Figure 4. An AI-based CT positioning system evaluated in 220 COVID-19 patients reduced positioning time by 28%, improved centering accuracy from 92% to 99%, reduced radiation dose by 16%, and decreased peripheral lung image noise by 9% [69]. DL approaches have also shown feasibility for landmark detection across positioning tasks [70,71]. In mammography, CNN-based systems achieved positioning accuracies of 96.5% for Cranio-Caudal (CC) views and 93.3% for Medio-Lateral Oblique (MLO) views in single institution evaluations [72], while additional DL methods reported true positive rates of 91.4% and 95.11% for positioning assessment [73,74]. The AI used in breast density estimation has also shown potential for improving interpretive consistency [75]. AI methods are also applied to dose and contrast optimization by incorporating patient characteristics into acquisition parameter selection [76,77]. This is particularly relevant in pediatric imaging due to higher radiation sensitivity [78]. Reported implementations include a CNN generating full-dose-equivalent brain MRI from low-dose gadolinium [79], a conditional GAN reducing double-dose perfusion [80], DLIR improving pediatric CT at lower dose [81], and combined DL reconstruction achieving 50% contrast and 60% radiation dose reductions in pediatric Coronary CT Angiography (CCTA) [82]. Operational risks include automation bias in atypical cases [64], alert fatigue requiring workflow configuration and human override [57], and performance variability across vendors and protocols, limiting transferability. Across the reviewed corpus, studies include single-center deployments [69,72], retrospective modality-specific evaluations [79,80,81,82], and feasibility studies in positioning tasks [70,71]. External validation across heterogeneous scanners and populations remains limited. Reported limitations include lack of external replication [69], protocol-dependent performance in pediatric dose reduction [81,82], sensitivity to acquisition parameters in contrast synthesis [79,80], and absence of prospective multi-site evaluations linking AI positioning to reduced repeat exams [76,77]. A further clinical risk specific to generative reconstruction and denoising models concerns the potential suppression of diagnostically relevant detail. Smoothing operations that reduce noise may simultaneously attenuate fine anatomical structures or low-contrast lesions, while synthesis-based approaches risk generating plausible but anatomically inaccurate tissue representations that obscure real pathology. These risks are inconsistently addressed in the reviewed literature and represent an important gap in the clinical validation of AI-based image processing at this stage. Table 5 summarizes key contributions and limitations. Evidence indicates heterogeneous maturity, with dose optimization approaching early clinical deployment (TRL 5–6), while automated positioning and protocol selection remain at feasibility level (TRL 3–4) [25,26,31].

### 3.5. Image Acquisition and Post-Processing

Image acquisition requires balancing radiation dose, scan duration, and diagnostic image quality under routine clinical constraints. Low dose protocols and patient motion frequently introduce noise and artifacts that reduce image interpretability and increase repeat examinations [84]. These challenges are particularly evident in CT, MRI, and ultrasound imaging, where motion sensitivity and hardware constraints can further degrade image quality [84,85,86,87]. AI applications at this stage focus mainly on post-processing, denoising, artifact reduction, and image reconstruction. DL methods have been widely applied to noise suppression and resolution enhancement across imaging modalities [84]. In brain MRI [85], deep learning-based denoising reconstruction (dDLR) demonstrated improved noise suppression and preservation of image quality compared with Denoising Convolutional Neural Network (DnCNN) and SCNN (Shallow Convolutional Neural Network) under the evaluated acquisition conditions. The Content-Noise Complementary Learning (CNCL) framework jointly models image content and noise using a generative adversarial network, reporting improved visual quality metrics and generalization across the evaluated datasets [86]. In ultrasound imaging, DL in real-time denoising systems have been evaluated for compatibility with live clinical workflows [87]. Additional approaches [88] include DL frame interpolation in respiration correlated Cone Beam Computed Tomography (CBCT) to reduce streaking artifacts [89] and artifact correction methods in CCTA that improved interpretability metrics for coronary artery disease assessment in the evaluated cohorts. Reconstruction approaches have been reported. DL reconstruction in neuroradiology showed acquisition time reductions up to 85% under specific protocols [90]. DLIR in low energy virtual monoenergetic imaging for dual-energy CT improved image quality while preserving diagnostic performance in rectal cancer staging [91]. These applications span artifact correction, noise suppression, contrast reduction, and reconstruction, with improvements in patient safety and image quality [67]. Operational considerations include the gap between image quality metrics and clinically validated outcomes. Gains in signal-to-noise ratio or sharpness do not necessarily improve lesion detectability or diagnostic confidence, and this distinction is inconsistently addressed [84,92]. Reconstruction models are sensitive to protocol variation and scanner differences, limiting transferability when trained on single-vendor data [85,90]. Real-time denoising in ultrasound and CBCT introduces computational latency that must be balanced with workflow integration [87,89]. Across the reviewed corpus, study designs are predominantly retrospective and single-center [85,87,88,89], with modality specific validation [86,90,91] and integrative reviews [92]. Prospective multi-center evaluation across heterogeneous hardware remains limited. Reported limitations include protocol dependence [85,90], computational cost [86], restricted cross-platform validation in ultrasound [87], sensitivity to acquisition parameters [88], and lack of outcome-level validation linking image quality to diagnostic gains [84,92]. Table 6 summarizes contributions and limitations. Evidence suggests these methods are approaching early clinical deployment, although outcome-level validation remains limited, corresponding to TRL 5–6 [25,26,31].

### 3.6. Radiologist Assignment and Worklist Prioritization

**Figure 5:**
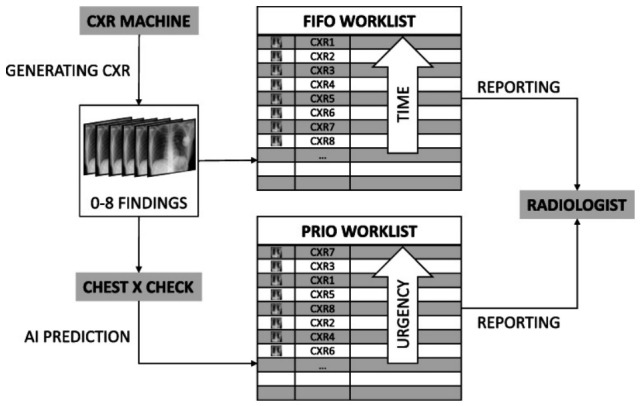
Workflow simulation illustrating AI-based worklist prioritization for chest X-ray (CXR) reading. CXRs are sorted into the worklist either chronologically (first-in, first-out (FIFO)) or by AI-predicted urgency (PRIO), and subsequently processed by a virtual radiologist [95]. CC BY 4.0.

Radiologist assignment and worklist prioritization determine the order in which studies are interpreted, coordinating urgency handling, case distribution, and reading workload to shape downstream reporting timelines and communication of critical findings. AI applications at this stage primarily function as triage and prioritization tools, screening incoming studies for suspected urgent findings and elevating them within radiologist worklists to reduce diagnostic delays [93,94,95,96]. AI for triage has been applied across head CT, chest radiography, CT pulmonary angiography (CTPA), and stroke imaging to stabilize reading order during backlog conditions and support timely escalation of time-sensitive findings, as illustrated in Figure 5. In acute neurovascular imaging [97], AI in Large Vessel Occlusion (LVO) detection was associated with a 22-min reduction in CT angiography to team notification time and a 23-min reduction in door to arterial puncture time compared with standard care in the evaluated cohort. Similarly, Intracranial Hemorrhage (ICH) AI integration reduced median time to detection from 512 to 19 min in a real world deployment study [98]. A systematic review of DL applied to worklist triage across ICH, LVO, pulmonary embolism, and pneumothorax reported pooled sensitivities and specificities above 80%, with measurable TAT reductions across conditions within the included studies [99]. For CTPA, AI prioritization was associated with a reduction in median time to diagnosis of incidental pulmonary embolism from 7714 min to 87 min, with a decrease in radiologist miss rates from 44.8% to 2.6% in a single-institution oncology cohort [100]. A separate evaluation of 11,252 CTPA examinations reported reduced TATs for PE-positive cases, with effects dependent on local workflow configuration [101]. Operational considerations include alert fatigue from false-positive prioritization, which may reduce responsiveness to escalation signals [93,95]. Performance is sensitive to disease prevalence, as models trained on high-prevalence cohorts may produce excess false positives in lower-prevalence settings, requiring threshold recalibration [94,96]. PACS integration and workflow configuration also influence TAT gains, with benefits in some settings not generalizing across institutions [99,101]. Across the reviewed corpus, study designs include retrospective analyses [94,96,100,101], simulation evaluations [95], prospective deployment studies [98], and systematic reviews [99]. No prospective multi-site randomized evaluation of integrated AI worklist prioritization has been reported. Reported limitations include prevalence sensitivity affecting false-positive rates [94,96], workflow-dependent gains [101], reliance on simulation rather than prospective validation [95], and heterogeneous study designs limiting interpretation [99]. Table 7 summarizes contributions and limitations. Evidence suggests these systems are approaching early clinical deployment for time-sensitive conditions, although false positives and workflow-related variability remain constraints for broader adoption, corresponding to TRL 6–7 [25,26,31].

### 3.7. Image Analysis and Interpretation

Image analysis and interpretation represent the most cognitively intensive stage of the radiology workflow, requiring visual attention, pattern recognition, and quantitative reasoning across modalities including CT, MRI, PET, ultrasound, X-ray, and mammography [102,103,104,105]. DL methods are widely applied to abnormality detection, structural delineation, biomarker quantification, and longitudinal analysis, with strongest performance in narrowly defined, high-volume clinical tasks [106,107]. This stage accounts for the largest share of published work and research activity within the radiology AI literature, reflecting its central role in diagnostic decision-making [14,105,108,109]. In neuroimaging, DL CAD systems applied to MRI and PET report diagnostic accuracies between 82% and over 95% for Alzheimer’s disease, Parkinson’s disease, and schizophrenia [110]. Multimodal MRI-PET approaches enable early Alzheimer prediction but remain limited by data standardization and generalisability [103]. In neurovascular imaging, a systematic review reported pooled sensitivity and specificity of 0.90 for aneurysm detection, with high risk of bias and limited external validation [14]. In cardiovascular imaging, AI supports biometrics extraction and phenotyping in echocardiography and cardiac MRI, reducing inter-observer variability [108]. ML and DL models achieve expert-level performance for coronary artery disease and cardiac amyloidosis detection [111,112], while multimodal risk models report AUC up to 0.964 but face interpretability and calibration challenges [105,113]. In pulmonary imaging, ensemble chest X-ray models achieve AUC up to 0.969 for COPD detection [114,115], whereas pulmonary hypertension classification remains sensitive to protocol variation [109]. Generative CT synthesis has been explored but remains limited by overfitting [116]. In musculoskeletal imaging, DL knee osteoarthritis grading achieved kappa 0.83 [117] and CT sarcopenia estimation reported AUC 0.85 [118]. In pediatric imaging, pneumonia detection approaches show near expert performance [35], while applications remain constrained by limited datasets and domain shift, with Tuberculosis detection AUC 0.697 [119] and pneumonia AUC up to 0.8464 [120,121]. In oncology, Lung cancer screening on low-dose CT achieved AUC 94.4% and sensitivity 94.6% [36]. Mammography models reduce false positives and negatives as second readers [34], although dataset diversity remains limited [122]. Despite strong performance, translation to clinical practice remains constrained. Accuracy in curated datasets does not necessarily generalize to heterogeneous environments [102,106]. Architectural complexity also introduces practical deployment constraints. Transformer-based models and LLMs require substantially greater computational resources than convolutional architectures, with inference latency and hardware prerequisites that may conflict with real-time clinical workflow demands, particularly in high-throughput or resource-limited settings where processing time directly affects reporting turnaround. Dataset bias persists in pediatric imaging [119,120] and oncology [123], as well as gender imbalance affecting performance [124]. Interpretability and calibration remain unresolved challenges [105]. Across studies, designs include retrospective single-institution evaluations [36,108,111,112,114,117,118], multi-center validation [34,103], and exploratory modeling [105,116]. Prospective multi-institutional validation remains limited. Key limitations include bias and limited external validation [14], data standardization challenges [103], interpretability gaps [105], protocol sensitivity [109], domain shift [119,120,125], demographic bias [126], and dataset limitations [122,127]. Overall, performance metrics are largely derived from retrospective or single-institution datasets, and may not generalize across heterogeneous environments. The evidence indicates heterogeneous maturity, with narrowly defined detection tasks approaching early clinical deployment (TRL 6–7), while broader disease modelling remains at the feasibility stage (TRL 3–4) [25,26,31]. Table 8 summarises representative contributions and key limitations across the reviewed diseases.

### 3.8. Report Generation and Communication

**Figure 6:**
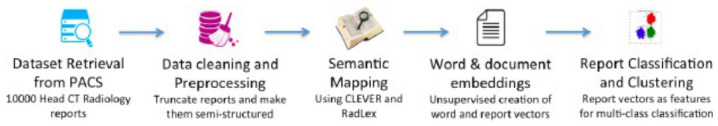
Intelligent Word Embeddings of Free Text Radiology Reports [131]. AMIA Open Access.

The integration of AI and NLP into radiology reporting aims to improve documentation efficiency and communication quality in a stage where reports remain largely unstructured and variable in terminology and format [128,129,130,131]. AI methods increasingly transform free text and image derived outputs into structured clinical narratives [132]. Most reported applications restrict AI to assistive roles—terminology standardization, structured extraction, templated drafting, and communication support rather than autonomous impression generation, as illustrated in Figure 6. The Intelligent Word Embeddings (IWE) method applied to chest CT automated pulmonary embolism characterization and improved inter-observer agreement [131], with a later multicentre extension reporting similar findings across institutions [133]. Subsequent work explored CNN and RNN (Recurrent Neural Network) pipelines [134], hierarchical LSTM (Long Short-Term Memory) with attention mechanisms [135], and transformer report generation [136]. Additional architectures incorporated contrastive attention [137], graph augmentation [138,139], and multi-view fusion [140]. Multimodal strategies combined imaging and clinical information through image plus history models [141], multimodal attention RNNs [142], retrieval generation agents [143], and CNN RNN systems [144]. Large scale pretraining enabled automated labeling and extraction of oncologic and disease-specific findings across evaluated report corpora [129,145,146,147]. More recent studies have examined generative AI and LLMs for report drafting, summarisation, and patient-facing explanations. GPT-2, GPT-3.5, and GPT-4 improved fluency and readability, although diagnostic impressions produced by these systems remained inferior to radiologist-authored reports and required human verification in evaluated settings [16]. GPT-4 has also been explored for knowledge synthesis [17], while ChatGPT-style systems have been assessed for drafting assistance and patient-oriented explanations [148,149]. Domain-adapted models such as ChatRadio-Valuer [150], RaDialog [151], and in-context alignment strategies [152] aim to improve clinical grounding and multimodal coherence. Fine-tuned LLMs have also been evaluated as proofreading tools capable of flagging reporting errors before sign-off [153]. Emerging agentic AI architectures enabling multi-agent coordination across workflow stages have also been described [154]. Operational considerations highlight three concerns. First, automation bias and alert fatigue may arise when fluent but inaccurate LLM outputs are presented without adequate verification [16,155]. Second, domain shift and limited external validation may reduce performance across different institutional reporting styles or disease distributions [130,133,145]. Third, reliable deployment requires vendor-neutral integration, transparent model provenance, and clearer medico-legal accountability, which remain incompletely addressed [128,132,155]. Fourth, large language models are susceptible to hallucination, whereby generated outputs may be clinically plausible in structure and language but factually incorrect or unsupported by the underlying imaging data. In radiology reporting, hallucinated findings carry direct patient safety implications, as erroneous descriptions of findings or impressions may propagate into clinical decision-making without detection if radiologist verification is incomplete. Across the reviewed corpus, study designs include retrospective NLP evaluations on curated report corpora [134,145,146,147], architecture development and benchmark comparisons [135,136,137,138], LLM feasibility and readability assessments [16,17,148,149], domain-adapted model evaluations [150,151,152], and emerging agentic system descriptions [154]. Reported limitations include inferior diagnostic impression quality in LLM-generated reports [16], cross-domain generalization gaps in NLP extraction models [130,133], task scope limiting integration [134,145,146], limited interpretability and external validation in domain adapted systems [150,151], and unresolved governance requirements for agentic architectures [154]. Evidence supports AI in this stage primarily as a reporting and communication co-pilot rather than an autonomous agent [132,156], corresponding to TRL 5–6 [25,26,31]. Table 9 summarises representative contributions and key limitations.

### 3.9. Follow-Up and Patient Engagement

Beyond image interpretation, radiology reports are often difficult for patients to understand due to technical terminology, inconsistent formatting, and limited translation of findings into actionable guidance. These barriers contribute to gaps in health literacy and shared decision making in the post-reporting stage [128,157]. AI applications aim to address these challenges by translating technical findings into more accessible language and supporting follow up communication. Current systems function as communication adjuncts rather than replacing clinician patient interaction, with use cases including report simplification, impression summarisation, and explanatory support under physician supervision. LLM tools have been evaluated in multi-center settings. GPT 4 systems applied to oncologic imaging reports improved patient comprehension and reduced consultation time under physician oversight [158]. The PRECISE framework also improved readability and patient satisfaction through structured, patient centred report reformulation [159]. Other approaches extend communication beyond text. ReXplain introduced avatar based video explanations with favourable user feedback, though validation remains limited to pilot settings [160]. ImpressionGPT has been evaluated for impression summarisation on large chest X ray datasets, reporting efficiency gains relevant to follow up workflows [161]. Comparative studies suggest that open weight LLMs may achieve performance comparable to proprietary models for simplification tasks while offering improved data privacy [162]. Operational considerations limit readiness for routine deployment. Simplified or generative outputs may omit clinically relevant details or introduce inaccuracies, creating misinterpretation risk without physician mediation [163]. Structural challenges include the absence of standardised metrics for patient understanding and limited longitudinal evidence linking AI mediated communication to clinical outcomes [157]. Reliance on proprietary platforms introduces interoperability challenges, vendor lock in, and uncertain medico legal accountability when integrated with health records and patient portals [132,157]. Across the reviewed corpus, study designs include multi-center LLM simplification evaluations [158,159], pilot and single institution studies [160,161], comparative privacy focused model assessments [162], and governance and risk analyses [157,163]. No prospective longitudinal evaluation linking AI-assisted communication to downstream clinical outcomes has been reported. Reported limitations include misinterpretation risk without physician mediation [163], pilot level validation [160], lack of standardised comprehension metrics [157], vendor lock in and governance constraints [132,157], and absence of prospective multi-site evaluations linking AI-assisted communication to longitudinal outcomes [158,159]. Table 10 summarises representative contributions and key limitations. Overall, evidence indicates that AI applications for follow-up communication and patient engagement remain at the feasibility stage, with most studies limited to early evaluations rather than prospective multi-institutional validation, corresponding to TRL 2–3 [25,26,31].

## 4. Cross-Stage Interoperability, Workflow Dependencies, and Maturity Assessment

Although AI applications across the stages are discussed individually in the preceding subsections, the radiology workflow functions as an interdependent system in which outputs from upstream stages influence the operational conditions under which downstream systems operate [21]. Consequently, limitations or variability introduced at one stage may influence the performance environment of subsequent stages. Understanding these cross-stage dependencies is therefore important when evaluating the real world operational impact of AI integration in radiology services. Errors or inefficiencies at Stage 1 (clinical ordering) influence downstream processes such as Stage 3 (scheduling) and Stage 4 (protocoling). Incomplete or poorly structured referrals may degrade the performance of NLP-based protocol selection tools, as documentation quality has been consistently identified as a limiting factor across ordering and protocoling applications [21,67]. Scheduling instability caused by no-show prediction failures at Stage 3 may also affect downstream acquisition conditions at Stage 5. Compressed acquisition windows in high-throughput environments can increase the likelihood of motion artifacts or repeat examinations, creating conditions that denoising and reconstruction models must subsequently address [58,84].

The relationship between Stage 6 (worklist prioritization) and Stage 7 (image analysis) illustrates another important workflow interaction. AI triage systems that generate excessive false-positive escalations may contribute to alert fatigue among radiologists, potentially reducing the efficiency gains that Stage 7 detection systems are designed to support [93,95]. In high-volume settings, prioritization imbalances may even affect time to diagnosis for genuinely urgent cases. Downstream dependencies are also evident. Structured data extracted during Stage 7 influences the performance of NLP generated reports at Stage 8. Models trained on curated outputs verified by radiologists may degrade when supplied with ambiguous or incomplete AI-generated annotations [130,132]. Report clarity at Stage 8 also affects the reliability of patient engagement tools at Stage 9, where LLM-based simplification systems depend on well-structured reports to generate safe and accurate patient-facing explanations [158,163]. Agentic AI architectures capable of coordinating outputs across multiple workflow stages have recently been proposed as a potential response to these dependencies [154]. However, governance frameworks, interoperability standards, and prospective validation of such systems remain underdeveloped at the time of writing [24,25]. Preliminary institutional models, including rubric driven evaluation frameworks proposed in recent work [164], suggest that structured multidisciplinary oversight may support more transparent and reproducible AI deployment decisions, although prospective multi-site validation remains limited. Cross-stage integration therefore represents a major operational opportunity and one of the least validated areas in current AI radiology research, underscoring the need for evaluation frameworks that measure end-to-end workflow outcomes rather than isolated stage-level performance. Progress toward this integration requires advances along three dimensions. At the data level, shared structured representations such as standardized imaging metadata, clinical indication formats, and report ontologies are needed to ensure that outputs from one stage can be used reliably by downstream systems. Interoperability standards such as Fast Healthcare Interoperability Resources (FHIR), Health Level Seven (HL7), and Digital Imaging and Communications in Medicine Structured Reporting (DICOM SR) provide partial solutions but remain inconsistently implemented across institutions [24]. At the system level, AI tools must exchange outputs through vendor neutral application programming interfaces (APIs) with version controlled models and documented provenance to support auditing and monitoring. At the evaluation level, current benchmarks assess performance at the individual task or stage level, whereas cross-stage integration requires frameworks that measure end-to-end workflow outcomes, including TAT, escalation accuracy, report completeness, and patient communication quality.

The maturity assessment presented is based on qualitative synthesis of the reviewed studies and reflects patterns observed within the included literature rather than a comprehensive or standardized evaluation of all existing AI systems, including proprietary or commercial deployments. Commercial implementation data is often not publicly available, limiting the feasibility of exhaustive assessment. Across the nine workflow stages, the available evidence reveals a clear maturity gradient with direct implications for both clinical adoption and research prioritization. Stages 6 (worklist prioritization), 7 (image analysis and interpretation), and 8 (report generation and communication) demonstrate the strongest evidence base, supported by prospective deployment studies, systematic reviews, and multi-center evaluations. These stages can therefore be considered at an early deployment readiness level, although prospective multi-institutional validation remains limited. In contrast, Stages 1 (clinical ordering), 3 (scheduling), 4 (protocoling and dose optimization), and 5 (image acquisition and post-processing) occupy an intermediate tier. While retrospective evidence is substantial and several task-specific applications are approaching deployment, externally validated prospective studies remain sparse. Stages 2 (pre-approval and billing) and 9 (follow-up and patient engagement) remain at a feasibility level, with evidence derived predominantly from single-institution technical evaluations rather than validated operational outcomes. This gradient is not merely descriptive but has direct implications for practice. Healthcare institutions may prioritise adoption of tools within Stages 6–8, where a more established evidence base exists, while exercising caution in procurement decisions at Stages 2 and 9 due to limited validation. For the research community, these findings highlight pre-approval, billing, and patient engagement as critical underexplored areas requiring methodological and translational advancement. The maturity gradient across the nine stages maps onto the Technology Readiness Level (TRL) scale introduced in Section 2 [25,26,31]. Stages 6–8 (worklist prioritisation, image analysis, and report generation) correspond to TRL 6–7, supported by prospective deployment studies and multi-centre evaluations. Stages 1, 3, 4, and 5 reflect TRL 4–5, where feasibility is established but externally validated prospective evidence remains limited. Stages 2 and 9 correspond to TRL 2–3, characterised by early proof-of-concept evaluations without validated operational outcomes. Institutions may reasonably adopt tools at higher TRL levels while exercising caution at lower stages. TRL assignments are inherently context-sensitive and reflect aggregate evidence rather than definitive classification of any individual system [25,26,31].

## 5. Limitations and Evidence Gaps

This narrative review synthesizes published radiology AI applications across nine workflow stages. The available literature is predominantly derived from retrospective, single institution, or feasibility oriented studies, with limited prospective and externally validated evaluations. Variability in datasets, validation strategies, and implementation settings may therefore affect the generalizability of reported findings. Research coverage across the workflow remains uneven. Image analysis and interpretation (Stage 7) and reporting (Stage 8) account for the majority of studies, whereas administrative and coordination stages, including ordering, scheduling, billing, and patient engagement, are comparatively underrepresented. This imbalance reflects current research activity rather than differences in clinical importance. Additionally, the reviewed studies were not evaluated for compliance with patient-level data splitting protocols, a methodological requirement whose absence has been shown to produce optimistic performance estimates in medical imaging tasks [30]. Radiology-specific work on AI-assisted billing, pre approval (Stage 2), and patient engagement (Stage 9) remains particularly limited, with most studies reporting early technical feasibility rather than validated operational outcomes [46,52]. These stages are included to highlight emerging areas and research priorities rather than comparable maturity. In addition, potential sources of bias, including vendor or developer involvement, were not systematically assessed and may influence reported performance, particularly in single institution studies. This review does not assess regulatory approval status, procurement considerations, or post market evaluation of AI systems. Inclusion of a method should therefore not be interpreted as evidence of regulatory clearance or routine clinical deployment.

## 6. Challenges and Future Directions

Although AI systems have been investigated across multiple stages of the radiology workflow, their integration into routine clinical practice raises technical, operational, and governance challenges that extend beyond algorithmic performance [165]. Many current applications focus on discrete tasks, such as detection, prioritization, or reporting assistance. However, achieving meaningful workflow improvements requires coordination across multiple stages of the radiology pathway, which depends on institutional infrastructure, interoperability, and implementation context. Interoperability between AI applications and core radiology information systems, including EMR, RIS, and PACS, remains variable across healthcare institutions [25,166]. These systems were not originally designed for modular AI integration or bidirectional interaction with external algorithms. Data repositories are often governed by proprietary standards and heterogeneous APIs, which can result in isolated deployments and limited reuse of AI outputs across workflow stages. Differences in ontologies, metadata structures, and prioritization logic further increase integration complexity and may affect the interpretation of AI-generated outputs [167,168]. In addition, vendor ecosystems may restrict access to structured data or imaging outputs, limiting interoperability across platforms [169]. Although interoperability standards such as DICOM, HL7, and FHIR provide technical frameworks for integration, variability in implementation continues to affect reproducibility across environments [25,170,171]. A complementary standardization layer is provided by the Integrating the Healthcare Enterprise (IHE) AI Results (AIR) profile, which defines how AI-generated outputs are structured and consumed within PACS and RIS environments, enabling vendor-neutral integration of multiple AI algorithms into existing clinical infrastructure. As multi-vendor AI deployment becomes the operational norm in radiology departments, the AIR profile is emerging as a foundational standard for reconciling algorithmic heterogeneity with clinical workflow continuity, particularly across the interpretive and reporting stages examined in this review [25]. Most published AI applications address narrowly defined objectives [102,172], and the translation from controlled evaluation to routine clinical practice remains limited, with surveys indicating that AI performance is perceived as inconsistent by the majority of radiologists who have deployed it [173].

In practice, however, radiologists often interact with multiple AI systems simultaneously, and combining outputs from separate tools can introduce additional workflow complexity [174,175]. Reuse of intermediate AI outputs across downstream tasks is rarely described in the literature. Moreover, infrastructure for version control, performance monitoring, and safety auditing differs substantially across vendors and institutions, increasing governance complexity in multi-vendor environments [26,167,169,176], and independent post-deployment audit frameworks that go beyond regulatory approval remain underdeveloped for radiology AI applications [177]. Recent developments in LLMs introduce further considerations for clinical deployment. These systems may be sensitive to prompt formulation, domain mismatch, and the generation of fluent but potentially inaccurate outputs [178,179,180]. Ensuring explainability and traceability of model outputs remains an active area of investigation in clinical contexts [181]. Reported mitigation strategies include task-specific fine-tuning, restricted deployment scopes, and structured human oversight when integrating LLMs into clinical systems. A related but distinct safety concern applies to generative models used in image acquisition and post-processing, where noise suppression and synthesis-based reconstruction algorithms may attenuate fine anatomical structures or produce plausible but inaccurate tissue representations, with the potential to obscure clinically relevant pathology. Prospective validation frameworks that assess fidelity to true anatomical detail, rather than aggregate image quality metrics alone, remain an unresolved requirement for these systems. A critical but underaddressed barrier to AI adoption in radiology is the distribution infrastructure required to serve the large imaging datasets on which these systems depend. When inference depends on transferring DICOM series to external cloud endpoints, network latency becomes a primary constraint, particularly for worklist triage in Stage 6 where clinical value is measured in minutes [99,100,101]. A three-tier hybrid architecture addresses this: on-premises computing for latency-sensitive tasks; edge deployment on modality workstations for acquisition-phase inference; and cloud platforms for training and batch workloads. Time-critical pathways including intracranial haemorrhage, large vessel occlusion, and pulmonary embolism triage are natural candidates for on-premises or edge inference [97,98]. Sustaining this infrastructure requires container-based deployment, model versioning [25,176,177], and inference optimisation including quantisation and knowledge distillation [26].

Cybersecurity and adversarial robustness also represent important system-level concerns. DL models have been shown to be vulnerable to adversarial perturbations that can induce misclassification [182]. In addition, cloud services and API-mediated data exchange may expand the technical attack surface for protected health information [183,184]. As AI tools are incorporated into scheduling, prioritization, and protocoling processes, system disruptions may propagate across interconnected workflow components [185]. Imaging cybersecurity standards and post-deployment monitoring strategies are still variably described in the literature [176], and regulatory frameworks such as the EU AI Act introduce new obligations for transparency, human oversight, and post-market surveillance that will directly shape radiology AI deployment [186]. Future research should therefore prioritize prospective validation of AI systems across diverse patient populations and institutional settings. Evaluations should assess not only diagnostic performance, but also the impact of AI on workflow efficiency, inter-reader agreement, patient outcomes, and healthcare system performance across the full radiology pathway. Operational benchmarks including inference latency, integration cost and interface usability should also be incorporated to better assess clinical deployability beyond algorithmic performance metrics [25,26].

Multicenter collaborations using harmonized imaging protocols, high quality annotations, and outcome confirmed reference standards will be essential to improve reproducibility. Large-scale benchmarking initiatives and standardized reporting frameworks can further support transparency and comparability across studies [167,176]. Integration of AI systems into PACS, RIS, and broader clinical infrastructures, together with human AI interaction models designed for clinical usability, will be critical for real world implementation across both administrative and clinical stages of the radiology workflow [25,171].

Finally, ensuring explainability, continuous performance monitoring, and regulatory compliance will be necessary to build clinical trust. Ethical considerations including data privacy, equitable access, mitigation of automation bias, and fair representation across patient populations must remain central to the AI deployment [167,175,184,187]. Empirical evidence that DL models can encode and propagate racial identity from medical images across modalities, often when clinical experts cannot [188], and that gender imbalance in training datasets consistently degrades classifier performance for underrepresented groups [124], underscores that bias in medical imaging AI represents a systemic challenge requiring proactive detection and mitigation across the full model development pipeline [189,190]. While AI offers important opportunities to improve radiology workflow efficiency and diagnostic support, careful integration will be required as these systems transition from research settings into routine clinical practice. Beyond addressing these challenges, future research should also investigate how AI systems can be coordinated across multiple stages of the radiology workflow rather than evaluated as isolated tools. Frameworks that enable interoperability, shared data representations, and standardized workflow metrics may help translate task level algorithmic performance into measurable improvements in radiology operations. Developing such system level integration strategies will be essential for realizing the full potential of AI-enabled workflow optimization across the radiology pathway.

## 7. Conclusions

This narrative review examined published AI applications across a nine-stage radiology workflow framework encompassing both clinical and administrative processes. The evidence reveals a clear maturity gradient: worklist prioritisation, image analysis, and report generation are best supported by prospective and multi-center studies, while pre-approval, billing, and patient engagement remain at an early feasibility stage. This gradient has direct implications for procurement and research prioritisation—institutions may reasonably adopt tools at Stages 6–8 where evidence is stronger, while exercising caution at Stages 2 and 9 where operational validation remains limited. Beyond stage-level maturity, the review highlights a structural gap between task-level algorithmic performance and system-level workflow impact. Most published AI applications address narrowly defined objectives and are evaluated in isolation, without accounting for how their outputs interact with adjacent workflow stages. As demonstrated across the reviewed evidence, errors or variability introduced at upstream stages propagate downstream, affecting the conditions under which subsequent AI systems operate. This interdependence suggests that the principal opportunity for AI in radiology lies not in further optimising individual tasks but in coordinating AI tools across the full imaging pathway. Realising this potential will require three shifts in research practice. First, evaluation frameworks must move beyond single-stage performance metrics toward end-to-end workflow outcomes, including turnaround time, escalation accuracy, report completeness, and patient communication quality. Second, interoperability infrastructure including standardised data representations, vendor neutral APIs, and consistent implementation of FHIR, HL7, and DICOM SR must be developed to enable reliable cross-stage data exchange. Third, governance frameworks for multi-stage AI deployment, including transparency, human oversight, and post-market surveillance mechanisms, must be established before agentic and integrated systems can be responsibly adopted in routine radiology practice.

## Figures and Tables

**Table 1:** Correspondence between the proposed nine-stage radiology workflow and existing workflow frameworks. ✓ = stage explicitly addressed; **∼** = partially addressed within another stage; **-** = not covered. Stages marked ^†^ are absent or marginal in all prior frameworks and represent the primary scope extensions of the proposed model.

#	Stage (This Review)	[20]	[21]	[22]	[25]	[26]	[23]	[24]
1	Clinical Test Ordering & Exam Selection	✓	✓	✓	✓	✓	✓	✓
2	Pre-approval Process & Billing ^†^	**-**	**-**	**-**	**-**	**∼**	✓	**∼**
3	Scheduling & Patient Flow Management	✓	**∼**	**-**	**-**	**∼**	✓	✓
4	Exam Protocoling, Patient Positioning, & Dose Optimization	✓	✓	✓	✓	✓	**-**	✓
5	Image Acquisition & Post-processing	✓	✓	✓	✓	✓	**-**	✓
6	Radiologist Assignment & Worklist prioritization	✓	✓	✓	✓	✓	**-**	✓
7	Image Analysis, & Interpretation	✓	✓	✓	✓	✓	**-**	✓
8	Report Generation & Communication	✓	✓	✓	✓	✓	**-**	✓
9	Follow-up & Patient Engagement ^†^	**∼**	**-**	**∼**	**-**	**-**	**-**	**∼**
	Stages explicitly covered	6/9	5/9	7/9	5/9	6/9	3/9	7/9

**Table 2:** AI methods for clinical test ordering and exam selection (Stage 1).

Ref.	Modality	Workflow Contribution	Study Limitation (Methodological)	Algorithmic/Clinical Limitation (Technical)
[21]	General Radiology	AI extraction from unstructured referrals to support exam selection	Narrative review; no prospective clinical evaluation	Performance dependent on documentation quality; degrades with incomplete referrals
[40]	Multiple Modalities	Guideline-aligned CDS reducing inappropriate imaging	Single-institution; no controlled comparison group	Physician acceptance barriers; workflow integration not demonstrated
[32]	Multiple Modalities	NLP matching of free-text indications to appropriateness criteria (top-3 accuracy: 98%, 85%, 86%)	Retrospective retrieval evaluation; no deployment outcome measured	Retrieval performance declines substantially with vague or ambiguous queries
[41]	Multiple Modalities	Multicenter deployment (266,029 orders) improved structured indications (34.6%→67.3%) and scoring (30%→52%)	Pre–post design; no concurrent control group	48% of orders remained unscored; coverage gap not resolved
[42]	Cardiac Imaging	ML personalization (9572 patients); lower events when aligned (*p* = 0.0024, 0.0321)	Post hoc model development; not prospectively randomized	Not validated outside the PROMISE trial cohort; generalizability unconfirmed
[43]	Radiology	Prospective study identifying interoperability and governance barriers	Single-institution; qualitative design	Interoperability and organizational barriers limit scalable deployment

**Table 3:** AI methods for pre-approval and billing processes (Stage 2).

Ref.	Modality	Workflow Contribution	Study Limitation (Methodological)	Algorithmic/Clinical Limitation (Technical)
[46]	Craniospinal CT/MRI	NLP ICD-10 coding; sensitivity 0.88, specificity 0.80, F2 0.83 for top codes	Single institution; only 200 reports evaluated	Accuracy limited to top-5 codes; moderate inter-rater agreement (α = 0.39–0.63)
[55]	MRI reports	Fine-tuned LLMs (GermanBERT, flanT5) for ICD-10 coding	German-language dataset only; no external validation	Performance drops substantially beyond the most frequent codes
[52]	Radiology department	NLP pipeline identifying overdue follow-up recommendations with revenue recovery	Single institution; indirect billing outcome only	Revenue recovery not linked to coding accuracy; no error rate reported
[53,54]	Prior authorization	High overturn rates of denied authorization decisions; evidence of inequitable denial patterns	Policy analysis only; no prospective or interventional evaluation	Algorithmic denials may disproportionately affect vulnerable populations; equity risks not mitigated

**Table 4:** AI methods for scheduling and patient flow management (Stage 3).

Ref.	Modality/Setting	Workflow Contribution	Study Limitation (Methodological)	Algorithmic/Clinical Limitation (Technical)
[59]	Multi-modality radiology (MGH)	Logistic regression predicted no-shows for 54,652 EMR appointments (AUC 0.77)	Retrospective; single institution; no intervention outcome	Limited predictor set; no assessment of automation bias risk
[58]	Multi-modality outpatient imaging	Gradient Boosted Trees on 4.5 M appointments; retrospective AUC 0.93; prospective AUC 0.73	Single outpatient site; only 6-week prospective validation	Prospective AUC substantially lower than retrospective; model degrades under workflow disruption
[60]	Outpatient MRI	Predictive analytics reduced MRI no-shows via targeted outreach	Single institution; retrospective pre–post design only	Outreach effect not isolated from concurrent confounders
[61]	CT, MRI, ultrasound, radiography (MGH)	Elastic net ML predicted wait times and appointment delays across modalities	Single institution; retrospective RIS data only	Dependent on RIS data completeness; not validated across platform types
[62]	Radiology department (MGH/Harvard)	Continuous-learning AI framework adapting to scanner use, patient throughput, and technologist variability	Conceptual framework; limited prospective validation reported	Retraining requirements and behavior under prolonged workflow disruption not characterized

**Table 5:** AI methods for exam protocoling, patient positioning, and dose optimization (Stage 4).

Ref.	Modality	AI Method	Workflow Contribution	Study Limitation (Methodological)	Algorithmic/Clinical Limitation (Technical)
[83]	Pediatric CT	CNN for DL reconstruction	Up to 85% radiation dose reduction with preserved image quality	Single center; limited external validation	Generalization across scanner vendors not demonstrated
[81]	Pediatric Head CT	DLIR	Improved image quality and lesion detection from low-dose CT	Single center; protocol-dependent evaluation	Performance varies with acquisition protocol; not validated across dose levels
[79]	Brain MRI	Encoder-decoder CNN	Full-dose-equivalent MRI from low-dose gadolinium input	Retrospective; single acquisition protocol tested	Sensitive to acquisition parameters; risk of synthesis artifacts masking pathology
[80]	Brain MRI	Conditional GAN	Single-dose contrast replacing double-dose perfusion imaging	Limited validation cohort; no multi-site replication	Synthesis artifacts may obscure low-contrast lesions
[82]	Pediatric CCTA	DLIR	50% contrast and 60% radiation dose reduction	Small cohort; constrained acquisition protocol	Not validated across heterogeneous patient body habitus

**Table 6:** AI methods for image acquisition and post-processing (Stage 5).

Ref.	Modality	AI Method	Workflow Contribution	Study Limitation (Methodological)	Algorithmic/Clinical Limitation (Technical)
[85]	Brain MRI	DnCNN, SCNN, dDLR	dDLR improved denoising over DnCNN and SCNN	Single center; single acquisition protocol	Protocol-dependent performance; limited cross-vendor transferability
[86]	CT, MRI, PET	GAN with CNCL	Joint content-noise modeling improved image quality	No prospective clinical validation	High computational cost; real-time feasibility not demonstrated
[87]	Ultrasound	DL with low-rank modeling	Real-time denoising compatible with clinical workflows	Single device type evaluated	Generalizability across ultrasound manufacturers not validated
[89]	CBCT	DL frame interpolation	Reduced streak artifacts with lower radiation dose	Controlled acquisition settings only	Performance under clinical motion variability not assessed
[88]	CCTA	DL artifact correction	Improved interpretability for coronary artery disease	Retrospective; single-center	Sensitive to acquisition parameters; no prospective outcome validation
[90]	Brain/Spine MRI	DL reconstruction	Up to 85% acquisition time reduction with maintained diagnostic quality	Limited cross-platform validation	Sensitive to undersampling patterns; performance vendor-specific

**Table 7:** AI methods for radiologist assignment and worklist prioritization (Stage 6).

Ref.	Modality	AI Method	Workflow Contribution	Study Limitation (Methodological)	Algorithmic/Clinical Limitation (Technical)
[94]	Head CT	AI priority scoring	ICH prioritization reduced wait time 15.75→12.01 min	Retrospective; single center	Prevalence-sensitive; excess false positives in low-prevalence settings
[95]	Chest X-ray	CNN (ResNet-50)	Reduced TAT for critical findings in FIFO vs. PRIO simulation	Simulation only; no prospective deployment	Radiologist behavioral response to AI prioritization not measured
[96]	Emergency CT	CNN detection	Up to 96% reduction in time-to-diagnosis for urgent cases	Single center; no multi-site replication	Performance sensitive to local PACS and workflow configuration
[93]	Multi-modality triage	AI triage & notification	Early escalation of time-sensitive examinations	Variable PACS integration across sites	Alert fatigue risk under sustained high false-positive rate
[100]	CTPA	DL-based AI triage	IPE diagnosis time 7714→87 min; miss rate 44.8%→2.6%	Single institution; oncology cohort only	Generalizability to non-oncology or mixed-prevalence populations not assessed
[101]	CTPA	CNN-based AI triage	Meaningful TAT reduction across 11,252 exams	Single center; workflow dependent	TAT gains vary with local workflow configuration; not reproducible across institutions
[99]	ICH, LVO, PE, pneumothorax	DL systematic review	Sensitivity/specificity >80%; TAT reductions across conditions	Heterogeneous study designs; limited prospective data	Pooled estimates mask inter-site variability; prevalence sensitivity not consistently addressed

**Table 8:** AI methods for image analysis and interpretation across major disease domains (Stage 7).

Ref.	Modality and Disease	AI Method	Workflow Contribution	Study Limitation (Methodological)	Algorithmic/Clinical Limitation (Technical)
[110]	MRI (Neurodegenerative)	CNN-based DL	Alzheimer’s and Parkinson’s detection with accuracy up to 95%	Limited dataset size; single-institution annotations	Label quality depends on clinical diagnosis; limited generalizability across imaging protocols
[108]	Echocardiography (Cardiac)	CNN-based DL	Automated ventricular measurements reducing inter-observer variability	Dependent on image quality at acquisition stage	Performance degrades with poor acoustic windows; not robust to acquisition variability
[114]	Chest X-ray (COPD)	Ensemble DL	COPD detection with AUC up to 0.969	Requires multi-center validation	Sensitive to image acquisition variability across scanners
[117]	X-ray (Osteoarthritis)	Deep CNN (ResNet)	Knee osteoarthritis grading (kappa 0.83)	Single-modality retrospective evaluation	Sensitive to patient positioning and image quality at acquisition
[119]	Pediatric X-ray (TB)	Vision Transformer	Pediatric TB detection (AUC 0.697)	Small pediatric dataset; zero-shot evaluation design	Substantial domain shift between adult training data and pediatric inference
[34]	Mammography (Breast cancer)	Ensemble DL	Reduced false positives (1.2%) and false negatives (5.7%)	Limited dataset diversity despite multi-center design	Limited explainability; performance sensitive to protocol variability

**Table 9:** AI methods for report generation and communication (Stage 8).

Ref.	Modality	AI Method	Workflow Contribution	Study Limitation (Methodological)	Algorithmic/Clinical Limitation (Technical)
[133]	Chest CT	Intelligent Word Embeddings	Pulmonary embolism report standardization; improved inter-observer agreement	Narrow disease scope; evaluated on single report corpus	Not generalizable beyond pulmonary embolism; terminology standardization only
[136]	Chest X-ray	Memory-driven Transformer	Automated report drafting; BLEU-4 up to 0.220	Evaluated on single benchmark dataset only	BLEU score does not reflect clinical accuracy; limited cross-domain generalization
[145]	CT/MRI (oncology)	Deep NLP extraction	Oncologic finding extraction accuracy up to 94%	Retrospective; single institutional report corpus	Task-specific; not transferable across disease domains or reporting styles
[16]	Multi-modality	GPT-4 LLM	Improved drafting efficiency and readability	Preliminary assessment; small evaluation set	Diagnostic impressions inferior to radiologists; hallucination risk without verification
[154]	Multi-modality	Agentic multi-agent AI	Automated coordination across reporting workflow stages	Conceptual and early-stage description only	Governance unresolved; human oversight requirements and failure modes not defined

**Table 10:** AI methods for report transformation and patient communication (Stage 9).

Ref.	Modality	AI Method	Workflow Contribution	Study Limitation (Methodological)	Algorithmic/Clinical Limitation (Technical)
[158]	Oncologic imaging	GPT-4 text simplification	Improved patient comprehension and reduced consultation time in multicentre evaluation	No longitudinal follow-up; physician-mediated setting only	Risk of omitting clinically relevant detail without physician review
[160]	Multi-modality	Avatar-based explanation (ReXplain)	Multimedia explanations improving patient engagement	Pilot study; no controlled outcome validation	Patient comprehension not objectively measured; usability not assessed across literacy levels
[161]	Chest X-ray	Iterative LLM summarisation (ImpressionGPT)	Automated impression summaries supporting follow-up workflows	Single modality; no prospective validation	Summary quality not benchmarked against radiologist-authored impressions
[162]	Chest radiography	Open-weight privacy-preserving LLM	Simplification performance comparable to GPT-4o with improved data privacy	No prospective multi-site evaluation	Simplification accuracy not validated against patient comprehension outcomes
[163]	Oncology	LLM communication risk analysis	Identifies misinterpretation and medico-legal risks	Observational analysis only; no interventional design	Risk characterization not linked to measurable patient safety or outcome metrics

## Data Availability

No new data were created or analysed in this study. Data sharing is not applicable to this article.
